# Effects of Two Different Modes of Task Practice during Lower Limb Constraint-Induced Movement Therapy in People with Stroke: A Randomized Clinical Trial

**DOI:** 10.1155/2021/6664058

**Published:** 2021-02-01

**Authors:** Auwal Abdullahi, Naima Umar Aliyu, Ushotanefe Useh, Muhammad Aliyu Abba, Mukadas Oyeniran Akindele, Steven Truijen, Wim Saeys

**Affiliations:** ^1^Department of Physiotherapy, Bayero, University Kano, PMB 3011, Gwarzo Road, Kano, Nigeria; ^2^Department of Physiotherapy and Rehabilitation Sciences, University of Antwerp, Antwerp, D.R.312, 2610 Wilrijk, Belgium; ^3^Department of Physiotherapy, Muhammad Abdullahi Wase Teaching Hospital, Kano, Off Audu Bako Way, Nassarawa G.R.A, PMB, 3160 Kano, Nigeria; ^4^Lifestyle Diseases Research Entity, Faculty of Health Sciences, North-West University, South Africa

## Abstract

**Background:**

Constraint-induced movement therapy (CIMT) is used for the rehabilitation of motor function after stroke.

**Objectives:**

The aim of this study was to compare the effects of lower limb CIMT that uses number of repetition of tasks with the one that uses number of hours of practice.

**Method:**

The study was a randomized clinical trial approved by the Ethics Committee of Kano State Ministry of Health. Fifty-eight people with stroke participated in the study. Groups 1 and 2 performed daily 600 repetitions and 3 hours of task practice, respectively, 5 times weekly for 4 weeks. Motor impairment (primary outcome), balance, functional mobility, knee extensor spasticity, walking speed and endurance, and exertion before and after commencement of activities were assessed at baseline and postintervention. The data was analyzed using Friedmann and Mann-Whitney *U* tests.

**Result:**

The results showed that there was only significant difference (*p* < 0.05) in knee extensor spasticity (group 1 (median = 0(0), mean rank = 27.50); group 2 (median = 0(0), mean rank = 31.64)), exertion before commencement of activities (group 1 (median = 0(0.5), mean rank = 21.90); group 2 (median = 1(0.5), mean rank = 37.64)), and exertion after commencement of activities (group 1 (median = 1(1), mean rank = 20.07); group 2 (median = 1(0), mean rank = 39.61) postintervention in favour of the experimental group (group 1)).

**Conclusion:**

The group 1 protocol is more effective at improving outcomes after stroke.

## 1. Introduction

One of the significant activities of daily living (ADL) human beings carry out is mobility such as walking. The ability to walk is largely controlled by the central nervous system (CNS) in which the brain is an integral part [1–3]. This control is however impaired in conditions that affect the brain such as stroke in which motor function of the lower limbs is impaired [4, 5]. Thus, one of the most important goals in stroke rehabilitation is to help patients regain motor function of the lower limbs and consequently the ability to walk independently as much as possible in order to ensure the ability to carry out ADLs [6]. To improve walking ability after stroke, constraint-induced movement therapy (CIMT) is used [7, 8].

The CIMT is a movement rehabilitation technique used to improve motor function after stroke. It was initially invented to force the use of the deafferented forelimbs in monkeys [9], but this has since then been translated to improve motor function following stroke in humans [10–13]. The most important components of CIMT for both upper and lower limbs are mass task practice with the affected limb, constraint of the unaffected limb, and transfer package [7, 11–19]. CIMT has a very strong evidence base. It helps improve walking ability, walking speed and other gait parameters, movement quantity and quality, and neurophysiological functions of the brain [7, 18, 20–22]. It also increases the expression of Growth-Associated Protein 43 (GAP-43) and numbers of *Δ*FosB-positive cells which are some of the biomarkers that play important roles in neural cell proliferation and neural and synaptic plasticity [20]. Similarly, movement of the lower limbs is very essential to brain health since reduced mobility results in a decrease in neural stem cell proliferation capability and altered cell cycle [23]. The neural stem cells are the self-renewing and multipotent cells that generate neurons and glial cells in the embryonic and adult brains [24].

One of the problems with CIMT is the prescription of the appropriate intensity of task practice enough to induce neuroplastic changes and recovery of motor function. This is because traditionally in both upper and lower limb CIMT protocols, duration in hours of practice is used as the measure of intensity of practice. In the lower limb CIMT specifically, the duration for practice has been reported to range between a couple of minutes to six hours per day [8, 23–26]. A CIMT protocol that uses 6 hours of task practice is the original CIMT, whereas the one that uses a shorter duration is known as the modified CIMT. However, the CIMT protocol that uses duration of practice has been argued to be not straight forward as duration does not correspond to a specific number of repetitions [27]. In contrast, how many times tasks are practiced is an important driver of recovery of motor function. Thankfully, the number of repetitions required for this recovery has been reported both in animals and humans, and it ranges between 289 and 1000 repetitions per day [28–35]. In particular, the studies on upper limb showed that using number of repetitions of task practice as a measure of intensity is feasible and effective at improving outcomes such as motor function, real-world arm use, and upper limb self-efficacy [32–34]. Unfortunately, majority of CIMT protocols used hours of practice as the measure of intensity of tasks being practiced. When hours are used, it is difficult to know whether patients achieved the amount of practice required for recovery. Therefore, it is wiser to ask our patients to carry out specific number of task repetitions during CIMT instead of leaving this open ended. This will help them to carry out the required number of repetitions known to result in recovery. The aim of this study was to compare the effects of a lower limb CIMT protocol using number of repetitions and the one using number of hours of practice. The study hypothesis was as follows: there will be a significant difference between CIMT protocol using number of repetitions and the one using number of hours of practice. This is because the protocol using number of repetitions seems to be more efficient, specific, and easy to follow in upper limb CIMT [27, 36].

## 2. Materials and Method

### 2.1. Study Design

The study was a randomized clinical trial approved by the Research Ethics Committee of the Kano State Ministry of Health (MOH/Off/799/T.I/688). It was also registered with the Pan African Clinical Trial Registry (PACTR201806003363142). In addition, consent for participation in the study and the publication of the study results was obtained from the participants.

### 2.2. Sample Size Estimate

The sample size of the study was estimated using G Power software version 3.1 [37]. The parameters used to estimate the sample size were effect size = 0.4, power = 0.8, and alpha value = 0.05. However, since 52 is the minimum sample size required to obtain a significant effect, 6 (10% attrition rate) was added to make it 58. This sample size calculation was based on the primary outcome which is motor function measured using lower limbs Fugl Meyer. In addition, a moderate effect size (0.4) was used in order to have a larger sample size even though a previous study by Danlami and Abdullahi has obtained a large effect size of >0.6 [38].

### 2.3. Participants

The study participants were stroke patients. The inclusion criteria used were as follows: participants with hemorrhagic or ischemic stroke in any stage of stroke, who were ≥18 years old, with good cognitive ability (a score of ≥24 on minimental state examination), with asymmetrical stance (assessed by observing the participant's standing posture), with ability to stand and walk with minimal assistance assessed using functional ambulation category, and who have ≥15 degrees of active knee flexion in the affected limb in standing position. This is because people with stroke have an extensor synergy pattern in the lower limbs which could impair their ability to flex the knee [39]. However, participants with severe pain that could interfere with training and those with hemineglect indicated by a cutoff of <44 on the star cancellation test were excluded from the study [40]. The study settings were physiotherapy departments of Murtala Muhammad Specialists Hospital and Muhammad Abdullahi Teaching Hospital in Kano, Nigeria.

The recruitment of the participants was done consecutively by a trained therapist in each of the study sites. A simple random technique using sealed opaque envelopes was used to allocate the participants into groups 1 and 2. The period for recruitment and follow-up was between 16^th^ May 2018 and 4^th^ July 2018.

### 2.4. Intervention and Control

Participants in both groups performed the following tasks: stepping forward, backward stepping, side stepping, ball kicking, and stair climbing. However, group 1 performed each of the tasks 40 times per session (altogether 200 repetitions), three sessions (morning, afternoon, and evening) per day (altogether 600 repetitions), five days per week, and with constraint applied only during practice sessions for four consecutive weeks. 600 repetitions were used because results of previous studies showed that for motor recovery to be achieved, task repetitions in the range of 300 to 800 per day is required [28, 29, 32]. On the other hand, group 2 performed modified CIMT consisting of three hours of task practice per day, five days per week, and with constraint applied during practice sessions for four consecutive weeks. Participants and their caregivers in both groups were trained on the first day by a very well-trained therapist in each of the study centers who was blinded to the aim of the study on how to carry out the tasks, and they were then asked to perform the tasks, two times a week at home under the supervision of the trained caregivers. This means that the five days a week treatment periods were divided into two days a week whereby the participants carried out the tasks at home under the supervision of their caregivers and three days a week whereby the participants carry out the tasks in the clinic under the supervision of the trained therapist. However, participants in both groups did not receive any other therapy such as conventional therapy throughout the study period.

To constrain the unaffected limb during practice, participants were told not to use the unaffected extremity during the practice. This was achieved by asking the participants to keep the limb still with the foot flat at one point and the hip and knee in full extension. In addition, participants were blinded to what each one was doing by requesting that they do not discuss the treatment given to them with other participants to avoid contamination bias. Furthermore, from time to time, calls were put through to the patients' relatives to monitor how the patients comply with the intervention's protocols. A logbook was also used by the relatives to record compliance with the protocols. When compliance is >90%, it indicates high adherence with the protocol [41]. Participants were also seen three times in the clinic, and this also assisted with the monitoring of the compliance.

### 2.5. Study Outcomes

In this study, lower limb motor function was the primary outcome which was measured using lower limb Fugl Meyer (FM). The secondary outcomes were balance measured using the Berg balance scale (BBS), functional mobility measured using the Rivermead Mobility Index (RMI), knee extensor spasticity assessed using the modified Ashworth scale (MAS), walking speed measured using the Ten-Meter Walk Test (10MWT), and endurance measured using the Six-Minute Walk Test (6MWT). In addition, exertion before and after commencement of activities was measured using hard activity chart for rating perceived exertion [42]. The lower limb Fugl Meyer (FM) is a reliable, valid, and responsive measure of motor function following stroke [43, 44]. Its scores range between zero and 34. The Berg balance scale (BBS) measures balance during functional tasks, and it consists of 14 items [45]. A five-point ordinal scale from zero to 4 is used to rate each item, in which a score of zero indicates poor balance and four indicates good balance ability. The RMI consists of 15 items (one of the items is direct observation of the patients, whereas the remaining 14 items are self-reports by the patients) arranged in increasing difficulty and scored on a scale of zero to one [46]. A score of zero indicates that the patient is unable to complete the task, whereas a score of one indicates that the patient is able to complete the task. The MAS is a reliable and valid scale that is rated on an ordinal scale of zero to four [47]. The 10MWT measures walking speed in meters per second over a short duration, and it has been reported to have excellent reliability for comfortable and fast gait speeds and predictive validity [48]. The 6MWT is also a valid and reliable submaximal exercise test for assessing aerobic capacity and endurance [49]. The hard activity chart is similarly a valid and reliable instrument [50]. All measurements were carried out at baseline, two weeks, and four weeks after the commencement of the interventions by blinded assessors.

The data obtained in the study was assessed for normality using the Kolmogorov-Smirnov statistics. Data for the demographic characteristics of the study participants was analyzed using descriptive statistics. The data on the study outcomes was analyzed using intention to treat analysis. However, in case of missing data, the mean imputation method was used [51]. Since the data obtained was not normally distributed (*p* < 0.05), change from baseline was analyzed using the Friedman test (for between-group data) and differences between groups were compared using the Mann-Whitney *U* test. The level of significance was set at a level of significance of <0.05. Where there was a significant difference within group or change from baseline, the Wilcoxon signed-rank test was used for post hoc analysis. The level of significance for the multiple comparisons was set at *p* < 0.02 using Bonferroni adjustment obtained by dividing 0.05 by three (the number of tests). All data were analyzed using SPSS version 20. The study protocol was published earlier [52].

## 3. Results

**Figure 1:**
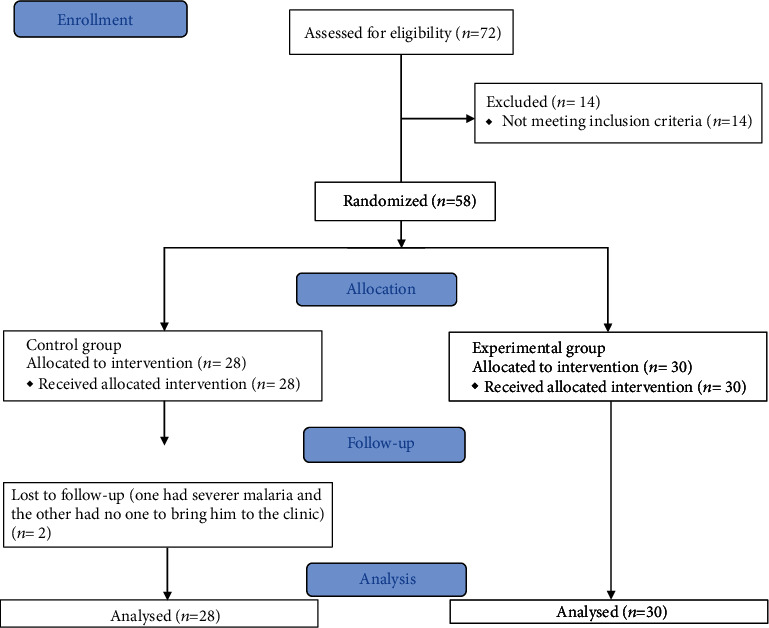
Study flowchart.

A total of seventy-two people with stroke were assessed for the study in which only 58 (80.5%) were eligible and participated in this study with age range, 18-75 years (30 participants in group 1 and 28 in group 2). The reason why there were 30 participants in group 1 but only 28 in group 2 was because we wanted to keep the number of participants in each group equal but because there were two hospitals where the data was collected, this resulted in an equal number of participants in the two groups. However, the study compliance was 96.5%; only two participants did not complete the study protocol (one had malaria and the other had no transport to the clinic). These participants were in group 2. In addition, only two participants (in group 1) reported mild low back pain and calf muscle pain, respectively. The characteristics of the study participants and the study flow chart are presented in Table 1 and Figure 1, respectively.

The results showed that, in both groups 1 and 2, there was significant improvement (*p* < 0.05) in the primary outcome (motor function) and the other study outcomes (balance, functional mobility, knee extensor spasticity, walking speed, endurance, and exertion before and after commencement of activities from baseline to 2 weeks and 4 weeks after the commencement of the intervention). Details of the results are presented in Table 2. For between-group comparisons, the results showed that there was only significant difference (*p* < 0.05) in knee extensor spasticity (group 1 (median = 0(0), mean rank = 27.50); group 2 (median = 0(0), mean rank = 31.64)), and exertion before commencement of activities (group 1 (median = 0(0.5), mean rank = 21.90); group 2 (median = 1(0.5), mean rank = 37.64)), and after commencement of activities (group l (median = 1(1), mean rank = 20.07); group 2 (median = 1(0), mean rank = 39.61) postintervention in favour of group 1). This is because the lower the scores for knee extensor spasticity and exertion, the better the improvement. Consequently, the mean ranks for group 1 are lower than those for group 2 in both knee extensor spasticity and exertion before and after commencement of activities. Details of the results are shown in Table 3.

## 4. Discussion

The aim of this study was to find out how effective is a lower limb CIMT protocol that uses number of repetitions of practice as a measure of intensity compared to a CIMT protocol that uses number of hours of practice at improving lower limb functional outcomes in people with stroke. The whole aim was to make therapy more efficient, make instructions to patients more specific, and try to increase therapy adherence by choosing one protocol. This is because the protocol using number of repetitions of practice seems to be easy to follow, specific, and efficient in upper limb CIMT [3, 36]. When a protocol is specific and easy to follow, compliance and adherence with the protocol are better achieved. Consequently, all the participants in the number of repetitions of the practice group completed the study, indicating that the protocol may be more feasible. In addition, only two participants (in group 1) reported mild low back pain and calf muscle pain, respectively, indicating that the protocol is safe. Serious adverse events can raise safety concerns and restrict the utility of an intervention. However, it is not significant in this case considering the mild nature of the pain and that only 6.7% of the participants in the group reported just a few adverse events.

Similarly, the results show that there is no significant difference between the two protocols in motor function, balance, functional mobility, walking speed, and walking endurance. Previous studies in upper limb CIMT using a protocol that uses number of repetitions of practice as a measure of intensity of practice reported it to be both feasible and effective at improving motor outcomes such as real-world arm use and motor function [32–34, 36]. In addition, the protocol takes less time (about one hour) to complete compared to the traditional or modified CIMT in which several hours are used for practice with no information on the intensity of tasks practiced. Thus, the protocol that uses number of repetitions of practice as a measure of intensity of practice can be used as an effective alternative for the one that uses number of hours of task practice as a measure of intensity of practice.

Studies using number of hours of practice in their protocols are thought to be not clear on the amount of the tasks practiced [27]. This is probably because of several reasons. One, in contrast with logic, short duration of task practice (<3 hours per day) has been shown to be more effective than long duration (≥3 hours per day) of task practice. Secondly, there is no certainty on whether the number of hours claimed are completely used for task practice. Report and analysis of previous studies indicated that only 3.95 hours out of 6 hours and 33% of the total time, respectively, were used for practice [53, 54]. However, the number of times tasks are practiced determines functional recovery [55], and in stroke literature, this number of repetitions has been determined [29–33]. According to Abdullahi, this number of repetitions can be achieved within one hour [27]. One hour of practice is far less than the time used in the traditional CIMT and many modified protocols of CIMT. This seems to suggest that it is not the time spent that matters, but how many times tasks are practiced. Thus, a CIMT protocol using number of repetitions of practice seems more desirable and easier to practice, whereas the CIMT protocol using number of hours of practice is time consuming. To the best of our knowledge, this study seems to be the only fully fledged RCT comparing a lower limb CIMT protocol that uses number of repetitions of practice with the one that uses number of hours of practice.

Furthermore, in the present study, knee extensor spasticity and exertion before and after the commencement of activity reduced more in group 1 than in group 2 at four weeks postintervention. Similarly, at two weeks postintervention, the reduction in exertion after commencement of activity has borderline significance in favour of group 1. This is an important finding since exertion can hinder task practice and delay walking recovery, as energy expenditure and cost during walking tend to be high in stroke patients [56]. However, according to Billinger and colleagues, consistent single limb exercise is an effective method for improving oxygen uptake and reducing energy expenditure during submaximal effort [21]. Therefore, the reduction in exertion in the present study which is better in group 1 could be explained by the virtue of the consistency in the exercise performance since participants had to practice the same number of repetitions of practice every day. Similarly, reduced spasticity in group 1 was noted in the knee extensors. Knee extensor spasticity is one of the sequelae of stroke, and improving this may be an important stroke rehabilitation milestone as it may help patients achieve independence in walking and other daily activities just like reduced exertion. Previous studies however showed that spasticity does not significantly contribute to walking dysfunction after stroke [57–59], but knee extensor muscles torque does [57]. The problems with these studies are that the sample size was small and those with spasticity were too small in number, respectively. Additionally, correlation does not mean cause and effect. In contrast, in the present study, spasticity and walking ability significantly improved after the intervention. This seems to suggest that spasticity may negatively affect walking ability and independence in patients with stroke.

Another issue in the study is feasibility of the group 1 protocol which was high, and interestingly, there were only a few adverse effects reported. In contrast, analysis of CIMT studies using number of hours of practice revealed that the compliance was not optimal [53, 54]. Although the authors could not give reasons for the nonoptimal compliance, this may be because of the demand of the protocol in terms of supervision, length of time, and lack of accountability in recording the intensity of practice. Thus, CIMT using number of task repetition may be a better alternative since task repetition can be counted even by the patients themselves [60]. However, the present study has some limitations too. One of the limitations is the majority of the participants (65%) in the study were in chronic stage of stroke (>6 months), and as such, the improvement in the outcomes of interest such as walking distance, functional ability, and balance might be as a result of compensation. Recovery of motor function is usually slow and small after 6 months poststroke [61–63]. Additionally, CIMT studies in chronic stroke patients usually combine CIMT with brain stimulation in order to achieve any improvement [64, 65]. Secondly, the time taken to perform the number of repetitions per session was not recorded, although previously, it was shown that 300 repetitions of task practice were possible within one hour [32]. In conclusion, the protocol of lower limb CIMT using number of repetitions of task practice is feasible and equally as effective as the one using number to hours of task practice. Therefore, number of repetitions of practice can serve as a suitable alternative of number of hours of practice since the intensity of task practice required for recovery of motor function is known when the former is used.

## Figures and Tables

**Table 1:** Baseline details on the study subjects.

Variable	Group 1 (*n* = 30)	Group 2 (*n* = 28)
Gender		
Male	12 (48%)	13 (52%)
Female	18 (44.6%)	15 (45.5%)
Age (years)	50.2 ± 13.9	47.8 ± 14.7
Stroke type		
Ischemic	25 (83.3%)	25 (89.3%)
Hemorrhagic	5 (16.7%)	3 (10.7%)
Limb dominance		
Right	28 (93.3%)	22 (78.6%)
Left	2 (6.7%)	6 (21.4%)
Side affected		
Right	20 (66.7%)	16 (57.1%)
Left	10 (33.3%)	12 (42.9%)
Time since stroke (weeks)	32 (80.5)^∗^	34 (70)^∗^
MMSE scores	27 (2)^∗^	28 (3)^∗^
FAC scores		
2	12 (40%)	10 (35.7%)
3	13 (43.3%)	14 (50%)
4	5 (16.7%)	4 (14.3%)
SCT scores	49 (3.25)^∗^	49 (4.75)^∗^
Knee flexion ROM (degrees)	32.5° (45)^∗^	49.5° (38.5)^∗^
Vital signs		
RR (cycles/min)	17 (5)^∗^	18 (4)^∗^
PR (beats/min)	89 (14)^∗^	82 (23)^∗^
SBP (mmHg)	130 ± 10.2	126.4 ± 15.3
DBP (mmHg)	80 (12)^∗^	85 (16.5)^∗^
Motor impairment	62.5 (8)^∗^	62 (9.5)
Balance	48 (5.3)^∗^	48 (5.5)^∗^
Functional mobility	12 (2)^∗^	12 (1)^∗^
Knee extensor spasticity		
0	11 (36.7%)	16 (57.14%)
1	15 (53.6%)	2 (7.14%)
2	4 (13.3%)	10 (35.71%)
Walking speed (m/s)	0.69 (0.59)^∗^	0.54 (0.58)^∗^
Walking endurance (meters)	141 (66.75)^∗^	134 (110.63)
Exertion		
0	8 (26.67%)	10 (35.71%)
0.5	6 (20)	9 (30%)
1	8 (26.67%)	7 (25%)
2	8 (26.67%)	1 (3.33%)
3	0 (0%)	1 (3.33%)

∗ = median and interquartile range; MMSE = minimental state examination; FAC = functional ambulation category; SCT = star cancellation test; ROM = range of motion; RR = respiratory rate; PR = pulse rate; SBP = systolic blood pressure; DBP = diastolic blood pressure.

**Table 2:** Within group differences in the study outcomes.

Variable	Group 1 (*n* = 30)					Group 2 (*n* = 28)				
	Baseline	2 weeks	4 weeks	*λ* ^2^	*p* value	Baseline	2 weeks	4 weeks	*λ* ^2^	*p* value
Motor impairment	62.5 (8)	63 (3)	64 (7)	50.634	<0.001	62 (10)	63 (9)	64 (8)	39.000	<0.001
Balance	48 (5)	50 (5)	51 (4)	51.869	<0.001	48 (6)	49 (7)	50 (7)	50.060	<0.001
Functional mobility	12 (2)	13 (1)	13 (2)	36.324	<0.001	12 (1)	13 (0)	13 (0)	29.778	<0.001
Knee extensor spasticity	1 (1)	0 (0)	0 (0)	32.500	<0.001	0 (2)	0 (1)	0 (0)	20.421	<0.001
Walking speed	0.69 (0.59)	0.63 (0.74)	0.66 (0.87)	41.862	<0.001	0.54 (0.57)	0.60 (0.64)	0.66 (0.71)	30.500	<0.001
Walking endurance	141 (66)	169 (90)	205 (117)	27.748	<0.001	134 (111)	138 (126)	226 (115)	40.775	<0.001
Exertion 1	1 (2)	1 (0.5)	0 (0.5)	32.109	<0.001	0.5 (1)	0.5 (0.5)	1 (0.5)	7.253	0.027
Exertion 2	2 (1)	2 (2)	1 (1)	24.930	<0.001	2 (2)	2 (1)	2 (0)	10.167	0.006

Exertion 1 = exertion before commencement of activities; exertion 2 = exertion after commencement of activities.

**Table 3:** Between-group differences in the study outcomes.

Time period	Variable	Group 1 (*n* = 30)	Group 1 (*n* = 28)	*U*	*p* value
Baseline	Motor impairment	62.59 (8)	62 (10)	356.000	0.321
	Balance	48 (5)	48 (6)	405.000	0.820
	Functional mobility	12 (2)	12 (1)	366.000	0.386
	Knee extensor spasticity	1 (1)	0 (2)	405.000	0.801
	Walking speed	0.69 (0.59)	0.54 (0.57)	417.000	0.963
	Walking endurance	141 (66)	134 (111)	385.000	0.586
	Exertion 1	1 (2)	0.5 (1)	321.000	0.111
	Exertion 2	2 (1)	2 (2)	347.000	0.236

2 weeks	Motor impairment	63 (7)	63 (9)	332.000	0.170
	Balance	50 (5)	49 (7)	370.500	0.438
	Functional mobility	13 (1)	13 (0)	397.000	0.698
	Knee extensor spasticity	0 (0)	0 (1)	387.000	0.450
	Walking speed	0.63 (0.74)	0.61 (0.64)	416.000	0.950
	Walking endurance	169 (90)	138 (126)	377.000	0.503
	Exertion 1	1 (0.5)	0.5 (0.5)	337.500	0.049
	Exertion 2	2 (2)	2 (1)	300.000	0.049

4 weeks	Motor impairment	64 (7)	64 (8)	331.000	0.166
	Balance	51 (4)	50 (7)	417.000	0.963
	Functional mobility	13 (2)	13 (0)	350.000	0.208
	Knee extensor spasticity	0 (0)	0 (0)	384.000	0.033
	Walking speed	0.66 (0.87)	0.66 (0.62)	406.500	0.834
	Walking endurance	205 (117)	226 (115)	303.500	0.070
	Exertion 1	0 (0.5)	1 (0.5)	137.000	<0.001
	Exertion 2	1 (1)	1 (0)	192.000	<0.001

Exertion 1 = exertion before commencement of activities; exertion 2 = exertion after commencement of activities.

## Data Availability

The data for the study is available on request to the corresponding author.

## Supplementary Materials

Supplementary Materials CONSORT 2010 checklist of information to include when reporting a randomized trial∗.
